# HOX transcription factors are potential therapeutic targets in non-small-cell lung cancer (targeting HOX genes in lung cancer)

**DOI:** 10.1038/sj.bjc.6604857

**Published:** 2009-01-20

**Authors:** L Plowright, K J Harrington, H S Pandha, R Morgan

**Affiliations:** 1 Postgraduate Medical School, Faculty of Health and Medical Sciences, University of Surrey, Surrey, UK; 2 Targeted Therapy Team, Chester Beatty Laboratories, The Institute of Cancer Research, London, UK

**Keywords:** lung cancer, NSCLC, HOX, PBX

## Abstract

The *HOX* genes are a family of homeodomain-containing transcription factors that determine the identity of cells and tissues during embryonic development. They are also known to behave as oncogenes in some haematological malignancies. In this study, we show that the expression of many of the *HOX* genes is highly elevated in primary non-small-cell lung cancers (NSCLCs) and in the derived cell lines A549 and H23. Furthermore, blocking the activity of HOX proteins by interfering with their binding to the PBX co-factor causes these cells to undergo apoptosis *in vitro* and reduces the growth of A549 tumours *in vivo*. These findings suggest that the interaction between HOX and PBX proteins is a potential therapeutic target in NSCLC.

The *HOX* genes are a family of homeodomain-containing transcription factors that determine the identity of cells and tissues in early development (Iimura and Pourquié, 2007), and also have key regulatory roles in adult haematopoietic stem cells and their descendants (Abramovich and Humphries, 2005). In addition, *HOX* genes are often overexpressed in malignant cells and are known to act as oncogenes in some haematopoietic malignancies (Eklund, 2007).

Repeated duplication events have given rise to 39 *HOX* genes in mammals, divided into four groups (A–D) in tightly linked clusters on different chromosomes (Hoegg and Meyer, 2005). The *HOX* genes are also divided into paralogue groups – genes that have the equivalent position in each cluster (1–13) – thus *HOXA1,* for example, is the 3′ most gene in cluster A (Scott, 1993). Whereas some *HOX* genes have distinct functions in specific contexts, many others have overlapping or redundant functions, both during early development (McIntyre *et al*, 2007) and in malignant cells (Eklund, 2007). This redundancy in HOX function is based in part upon the binding of similar DNA sequences and also on the interaction of HOX proteins with a common set of co-factors including PBX and MEIS. PBX and MEIS modify the DNA-binding specificity of HOX proteins, influence the regulation of transcription and are required for many aspects of HOX function (Moens and Selleri, 2006).

In addition to haematological malignancies, the *HOX* genes are also expressed at high levels in many other solid malignancies, although with the exception of melanoma (Morgan *et al*, 2007), they are not known to have an oncogenic function. A number of studies, focusing on small groups of *HOX* genes, have shown that some are upregulated in lung cancer (Abe *et al*, 2006), but the functional significance of this is unknown. Here we present a comprehensive analysis of *HOX* expression in non-small-cell lung cancer (NSCLC) that reveals that many of the *HOX* genes have strongly elevated expression in malignant cells. Further, we show that antagonising HOX/PBX binding in the NSCLC cell lines A549 (Lieber *et al*, 1976) and H23 (Little *et al*, 1983) induces apoptosis *in vitro* and causes significant tumour shrinkage in A549 nude mouse models.

## Materials and methods

### Maintenance of A549 and H23 in culture

The NSCLC cell lines A549 and H23 were obtained from the American Type Culture Collection (ATCC, Manassas, VA, USA). The cells were cultured in F-12K Nutrient Mixture Kaighn's Modification medium (Invitrogen, Paisley, UK) supplemented with 10% foetal calf serum (Invitrogen) and 1% penicillin (10 000 U ml^−1^)/streptomycin (10 mg ml^−1^) (Sigma, Gillingham, UK). Cell cultures were maintained at 37°C in a humidified, 5% CO_2_ incubator.

### Synthesis of HXR9 and CXR9 peptides

HXR9 is an 18-amino-acid peptide consisting of the previously identified hexapeptide sequence that can bind to PBX and nine C-terminal arginine residues (R9) that facilitate cell entry (Morgan *et al*, 2007). The N-terminal and C-terminal amino bonds are in the D-isomer conformation, which has earlier been shown to extend the half-life of the peptide to 12 h in human serum (Morgan *et al*, 2007). CXR9 is a control peptide that lacks a functional hexapeptide sequence and that includes the R9 sequence. All peptides were synthesised using conventional column-based chemistry and purified to at least 80% (Biosynthesis Inc., Lewisville, TX, USA).

### Imaging of cell cultures

Cells were plated in six-well plates in 2 ml of medium and allowed to recover for at least 24 h. When approximately 60% confluent, the cells were treated with the active peptide HXR9 (60 *μ*M) or the control peptide CXR9 (60 *μ*M) for 3 h. For phase-contrast micrographs, the cells were washed twice with cold PBS and visualised using a Nikon Eclipse TS100 inverted microscope and images recorded using a Nikon camera and capture software (Jencons).

### Analysis of cell death and apoptosis

Cells were treated with HXR9 or CXR9 as described above. Assessment of cell viability was performed using the MTS assay (Promega, Madison, WI, USA) according to the manufacturer's instructions. Cells were harvested by incubating in trypsin-EDTA (Sigma) at 37°C until detached and dissociated. Apoptotic cells were identified using flow cytometry (Beckman Coulter Epics XL Flow) and the Annexin-V-PE apoptosis detection kit (BD Pharmingen, Franklin Lakes, NJ, USA) as described by the manufacturer's protocol.

### RNA purification and reverse transcription

Total RNA was isolated from cells using the RNeasy Plus Mini Kit (Qiagen, Crawley, UK) by following the manufacturer's protocol. The RNA was denatured by heating to 65°C for 5 min. cDNA was synthesised from RNA using the Cloned AMV First Strand Synthesis Kit (Invitrogen) according to the manufacturer's instructions.

### Quantitative PCR

Quantitative PCR was performed using the Stratagene MX3005P real-time PCR machine and the Brilliant SYBR Green QPCR Master Mix (Stratagene, La Jolla, CA, USA). Oligonucleotide primers were designed to facilitate the unique amplification of *β-actin*, *c-Fos* and each *HOX* gene.

### Transcriptional profiling

Total RNA was extracted from A549 cells treated with CXR9 or HXR9 (60 *μ*M) for 3 h, and was used as a template to generate Cy3-labelled cRNA using the Low RNA Input Linear Amplification Kit (Agilent, Santa Clara, CA, USA). Each Cy3-cRNA was used as a probe on the Whole Human Genome Microarray (4 × 44K) slide. This microarray consists of 60-mer oligonucleotides with sequences representing more than 41 000 human genes. The microarray slides were scanned and data were extracted using the Agilent Feature Extraction Software (version 9.5.3). Data were analysed using GeneSpring GX software.

### Mice and *in vivo* trial

All animal experiments were conducted in accordance with the United Kingdom Co-ordinating Committee on Cancer Research guidelines for the Welfare of Animals in Experimental Neoplasia (Workman *et al*, 1998) and approved by the St George's Hospital Medical School Ethical Review Committee. The mice were kept in positive pressure isolators in 12 h light/dark cycles and food and water were made available *ad libitum*.

Athymic nude mice were inoculated subcutaneously with a suspension of 2.5 × 10^6^ A549 cells in culture media (100 *μ*l). Once tumours reached volumes of approximately 100 mm^3^, mice received an initial dose of 100 mg kg^−1^ CXR9 or HXR9 either intraperitoneally or intratumorally, with subsequent dosing of 10 mg kg^−1^ twice weekly. Each group contained 10 mice. The mice were monitored carefully for signs of distress, including behavioural changes and weight loss. Animals were euthanised if the tumour volume exceeded 400 mm^3^.

### Statistical analysis of the data

Data are given as means±s.e.m. of multiple independent experiments. Significant effects were determined using Student's *t*-test.

## Results

### *HOX* gene expression in NSCLC

**Figure 1:**
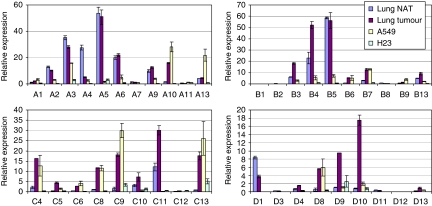
*HOX* gene expression in primary lung NSCLC tumours, normal adjacent tissue (NAT) and derived cell lines. The expression of each *HOX* gene was determined by Q-PCR and is presented as a ratio to the amount of *β-actin* transcript ( × 1000). Error bars show the s.e.m.

The HOX proteins of paralogue groups 1–9 bind the co-factor PBX and are thus potential targets of HXR9. To examine the expression of *HOX* genes in these paralogue groups, RNA was extracted from three separate cultures of A549 and H23 together with six primary NSCLC tumours retrieved during biopsy and corresponding normal adjacent tissue. Quantitative PCR was used to assess the relative numbers of *HOX* transcripts. Differences in transcription are apparent for many of the *HOX* genes (Figure 1). Notably, a number of genes in the *HOXA* group are strongly expressed in normal tissue, but are downregulated in both primary tumours and A549, including *HOXA3* and *HOXA5*. Conversely, many members of the *HOXC* and *HOXD* groups are strongly upregulated in the primary tumours and A549, including *HOXC4*, *HOXC8*, *HOXC9*, *HOXC13*, *HOXD8* and *HOXD10*. H23 cells also express *HOX* genes but to a considerably lesser degree than the other cell types studied.

### Disrupting the interaction between HOX and PBX triggers apoptosis in NSCLC cells

**Figure 2:**
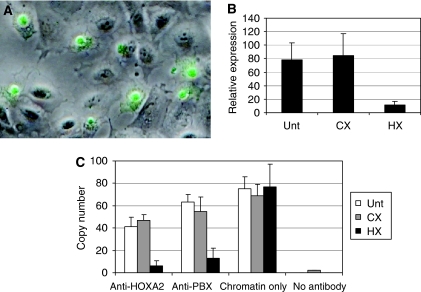
HXR9 blocks HOX/PBX dimer formation in NSCLC cells. (**A**) HXR9 uptake by A549 cells. A fluorescent micrograph of A549 cells incubated with 15 *μ*M HXR9-FITC (green) for 2 h, combined with the corresponding phase-contrast image. HXR9 is primarily located in the nucleus. (**B**) *HOXA2* expression is maintained in part through a HOX/PBX-binding site in its promoter. Correspondingly, Q-PCR analysis of H23 cells treated with 60 *μ*M HXR9 for 2 h reveals that *HOXA2* expression is significantly reduced upon HXR9 treatment. (**C**) ChIP analysis of the *HOXA2* promoter. Chromatin was extracted from HXR9- or CXR9-treated cells. After cleavage, fragments were immunoprecipitated using an anti-HOXA2 or an anti-PBX antibody, and the number of copies of the *HOXA2* promoter region recovered was determined by Q-PCR. A reduced number of *HOXA2* promoter sequences is recovered from HXR9-treated cells when immunoprecipitating with anti-HOXA2 or anti-PBX antibodies, whereas no significant difference is apparent when equal amounts of chromatin are used without selection.

The high levels of *HOX* expression in NSCLC together with the potential oncogenic function of *HOX* genes (Eklund, 2007) suggest that they may be a therapeutic target in this disease. However, the high level of functional redundancy exhibited by the HOX transcription factors, together with our observation that many different *HOX* genes are expressed, makes a conventional genetic knockdown approach difficult to use. For this reason, we chose to target the interaction between HOX proteins and the PBX co-factor. HOX/PBX dimers have an enhanced binding affinity and specificity for DNA and are able to activate or repress the transcription of a subset of target genes (Phelan *et al*, 1994; Piper *et al*, 1999; Morgan *et al*, 2000). Dimerisation is mediated by a six-amino-acid region found in the majority of HOX proteins (the ‘hexapeptide’ motif (Piper *et al*, 1999)). A peptide mimic of this region linked to a polyarginine sequence (that facilitates cell entry (Jiang *et al*, 2004)) might act as a competitive inhibitor of HOX/PBX binding. We therefore synthesised a peptide that consists of the hexapeptide sequence linked to nine arginine residues (HXR9) and a control peptide lacking a functional hexapeptide sequence (CXR9). Using a fluorescently tagged version of HXR9, we demonstrated that the peptide was taken up by NSCLC cells in culture and became concentrated in the nucleus within 2 h of application (Figure 2A).

To determine whether HXR9 can specifically disrupt HOX/PBX formation (and consequently DNA binding), we studied the effects of HXR9 on *HOXA2* expression. *HOXA2*, like a number of other *HOX* genes, is positively regulated by binding of HOX/PBX proteins to consensus sequences in the *HOXA2* promoter (Tümpel *et al*, 2007). Hence, if HXR9 can disrupt this interaction, it should reduce *HOXA2* expression, which it does (Figure 2B). To show that HXR9 actually prevents the interaction between HOX and PBX proteins we used chromatin immune precipitation analysis of HXR9- and CXR9-treated H23 cells. The presence of the HOX/PBX-binding site in the *HOXA2* promoter results in the enrichment of this sequence when chromatin is selected using either an anti-HOXA2 or an anti-PBX2 antibody. However, pre-treatment of cells with HXR9, but not CXR9, causes a significant reduction in the number of *HOXA2* promoter copies recovered using this technique (Figure 2C), indicating that HXR9 does indeed directly disrupt the formation of a HOX/PBX complex.

Given the high level of *HOX* expression in primary tumours and A549 and H23 cells, we investigated whether HXR9 could trigger cell death in a similar manner to that observed for melanoma cells (Morgan *et al*, 2007). Treatment of cultures with HXR9 (60 *μ*M) for 3 h resulted in a high degree of cell death whereas CXR9 had no discernable effects. The IC_50_ (24 h) for HXR9 was determined to be 32.5 *μ*M (s.e.m. 0.6 *μ*M) in A549 and 69 *μ*M (s.e.m. 1.2 *μ*M) in H23, whereas that for CXR9 was in excess of 200 *μ*M in both cell lines (data not shown).

**Figure 3:**
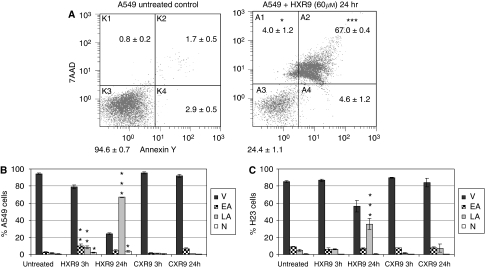
HXR9 triggers apoptosis in A549 and H23 NSCLC cells. Flow cytometric analysis of Annexin-V-PE-stained A549 and H23 cells demonstrated induction of apoptosis in cultures treated with HXR9. Data are the mean±s.e.m. of four independent experiments. ^*^P<0.05, significantly different from CXR9-treated cells; ^**^P<0.005, highly significantly different from CXR9-treated cells; ^***^P<0.0001, extremely significantly different from CXR9-treated cells. (**A**) Example plots of flow cytometric data for untreated and HXR9-treated A549 cells. *x* axis – Annexin-V staining indicating degree of apoptotic changes in the cell membrane; *y* axis – 7-AAD vital dye counterstaining as a measure of cell permeability. (**B**) Summary of flow cytometry data for A549 cells and (**C**) for H23 cells. Error bars represent the s.d. (*n*=4); ‘V’ – viable cells not in apoptosis, ‘EA’ – cells in early apoptosis, ‘LA’ – cells in late apoptosis and ‘N’ – necrotic cells.

To distinguish between apoptotic and necrotic death, cells were analysed using flow cytometric analysis after labelling with the vital dye 7-Amino-actinomycin D (7-AAD) or Annexin-V-FITC. 7-Amino-actinomycin D can enter only dead cells, whereas Annexin-V-FITC recognises changes in the plasma membrane that are characteristic of apoptosis (Koopman *et al*, 1994). A substantial number of A549 cells treated with HXR9 (60 *μ*M) were shown to be undergoing some form of cell death (Figure 3A and B). When compared with untreated or CXR9-treated cells, a significant proportion of HXR9-treated cells (*P*<0.005) were in either early or late apoptosis, or were necrotic (*P*<0.05). Similar changes were also observed for H23 cells (Figure 3C).

### HXR9 causes transcriptional changes

**Figure 4:**
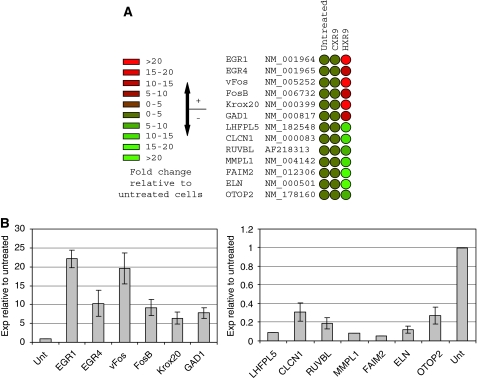
HXR9 causes transcriptional changes. (**A**) Subsequent to treatment with CXR9 or HXR9, RNA was extracted from A549 cells and analysed by microarray. There was no significant change in the transcription of the majority of genes, although five of them showed an increase, and eight a decrease, of five-fold or greater. (**B**) Semi-quantitative PCR was used to confirm the microarray findings. Gene expression was determined relative to *β*-actin expression and results are presented as a ratio with the expression of each gene in untreated cells. Error bars represent the s.e. of the mean.

To study the transcriptional changes that occur when HOX/PBX dimers are disrupted by HXR9 we profiled the transcriptome of CXR9- and HXR9-treated cells using a whole human genome microarray. RNA was extracted from A549 cells treated with 60 *μ*M HXR9 or CXR9 for 3 h and used as a template to generate Cy3-labelled cRNA for microarray screening. This revealed that a number of genes were highly upregulated, including a number that had earlier been observed in melanoma cells (Morgan *et al*, 2007). Most significant among these were v*Fos* and *FosB*, both of which were upregulated more than five-fold in HXR9-treated cells compared with controls (Figure 4A). The relative magnitude of these changes was confirmed using semi-quantitative PCR (Figure 4B).

### HXR9 blocks A549 tumour growth *in vivo*

**Figure 5:**
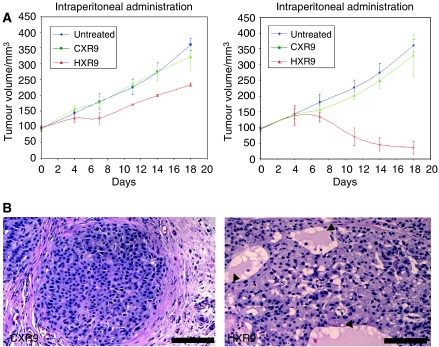
HXR9 blocks tumour growth *in vivo*. (**A**) Thymic nude mice were inoculated subcutaneously with 2.5 × 10^6^ A549 cells. When tumours reached volumes of approximately 100 mm^3^, the animals received an initial dose of 100 mg kg^−1^ of CXR9 or HXR9 either in to the peritoneum or intratumorally, with subsequent dosing of 10 mg kg^−1^ twice weekly. (**B**) Structural changes in HXR9-treated tumours. Tumours were excised from mice after the 18-day treatment cycle (twice weekly intraperitoneal administration of 10 mg kg^−1^ HXR9), embedded and sectioned. Distinct differences in tumour architecture are apparent in HXR9-treated mice, most notably in areas where extensive cell death has occurred (arrow heads). Scale bar: 100 *μ*m.

To establish whether HXR9 could block A549 tumour growth *in vivo*, A549 flank tumours were established in nude mice. When these had reached an average volume of 100 mm^3^, twice weekly treatments of HXR9 or CXR9 were administered by injection directly either into the tumour or into the peritoneum. After 18 days of treatment, the tumours of HXR9-treated mice were considerably smaller than those of the control groups (Figure 5A), and histological analysis revealed changes in tumour architecture consistent with widespread cell death (Figure 5B).

## Discussion

The *HOX* genes are well established as having oncogenic potential in a range of haematological malignancies (Eklund, 2007), and recent studies have indicated that they also allow cell survival in melanoma by blocking the transcription of pro-apoptotic genes (Morgan *et al*, 2007). Here we have shown that disrupting the interaction between HOX and PBX proteins is sufficient to cause apoptosis in the NSCLC-derived cell lines, A549 and H23. Although earlier studies have also shown HOX genes to be dysregulated in NSCLC (Abe *et al*, 2006), to our knowledge this is the first report to describe these changes in a comprehensive manner allowing a direct comparison between expression in normal lung tissue, primary tumours and derived cell lines. This reveals that there is a large increase in transcription in most *HOX* genes of the *HOXC* and *HOXD* families when comparing normal lung tumour with primary tumours, and furthermore, this change is also present in A549 and H23, indicating that there has been a stable change in the transcriptome.

Analysis of changes in transcription upon the addition of HXR9 revealed that the Early Growth Response 1 (*EGR1*) gene is upregulated by more than 20-fold. *EGR1* has been shown to mediate apoptosis in a number of malignancies (Pignatelli *et al*, 2003; Yu *et al*, 2007), possibly by activating the transcription of pro-apoptotic genes such as *PTEN*, and *EGR1* can also block angiogenesis and tumour invasion (Lucerna *et al*, 2006; Yu *et al*, 2007). Further, high levels of *EGR1* correlate with improved survival in NSCLC patients (Ferraro *et al*, 2005) and is upregulated when NSCLC cells are treated with the apoptosis-inducing retinoid 6-[3-(1-adamantyl)-4-hydroxyphenyl]-2-naphthalene carboxylic acid (Sakaue *et al*, 2001).

It is also noteworthy that HXR9-treated A549 cells show a large increase in the transcription of *vFos*, *cFos* and *FosB*. A similar response is apparent in melanoma cells in which a large increase in *cFos* transcription mediates the pro-apoptotic effect of HXR9 (Morgan *et al*, 2007). *cFos* and its binding partner *Jun* are both members of the bZIP superfamily of transcription factors, which are characterised by a basic DNA-binding domain combined with a leucine zipper region (Hess *et al*, 2004). JUN can homodimerise to form the AP-1 transcription factor, which activates the expression of a number of genes involved in cell cycle regulation, including *cyclin D1* (Albanese *et al*, 1995). However, an elevated *cFos* expression can also lead to apoptosis in hepatoma cells (Kalra and Kumar, 2004) and hepatocytes (Mikula *et al*, 2003). Furthermore, recent studies have shown that *cFos* transcriptionally represses the key antiapoptotic gene *c-FLIP*(*L*), greatly sensitising prostate cancer cells to TRAIL-induced apoptosis (Li *et al*, 2007; Zhang *et al*, 2007). Hence strategies to increase *cFos* transcription could be valuable in making malignant cells vulnerable to apoptosis, and HXR9 may synergise with TRAIL and other drugs that induce apoptosis to facilitate enhanced cell killing across a broad range of tumour types.
